# Neuromyelitis Optica: A Case Report From a Radiological Perspective

**DOI:** 10.7759/cureus.38945

**Published:** 2023-05-12

**Authors:** Zubir S Rentiya, Ogbonnaya Akuma, Madiha Haseeb, Chinwe C Okonkwo, Dr. Aadil Khan

**Affiliations:** 1 Department of Surgery, MedStar Georgetown University Hospital, Washington, USA; 2 Department of Radiation Oncology and Radiology, University of Virginia School of Medicine, Charlottesville, USA; 3 Department of Internal Medicine, Ebonyi State University, Abakaliki, NGA; 4 Department of Neurology, Dow University of Health Sciences, Karachi, PAK; 5 Department of Family Medicine, School of Medicine, Caribbean Medical University, Willemstad, CUW; 6 Department of Cardiology, University of Illinois at Chicago, Chicago, USA

**Keywords:** transverse myelitis, aquaporins-4, magnetic resonance imaging ( mri ), azathioprine, devic's neuromyelitis optica

## Abstract

Neuromyelitis optica (NMO), also known as Devic’s disease, is a chronic inflammatory disorder of the optic nerve and the spinal cord. Similar to multiple sclerosis, it has a relapsing and remitting characteristic. The disease is characterized by optic neuritis and longitudinal extensive inflammation of the spinal cord. Magnetic resonance imaging (MRI) is the modality of choice for this disorder. The serological examination also shows the presence of aquaporin-4 (AQP4) autoantibodies. MRI shows longitudinal extensive transverse myelitis and signs of optic neuritis such as inflammation of the optic nerve. The treatment is based on intravenous corticosteroids with or without plasmapheresis. The current case is a 25-year-old African American male patient who presented with multiple sclerosis-like symptoms (i.e., optic neuritis and transverse myelitis) but turned out to have NMO. Serological examination reveals the absence of AQP4 autoantibodies. A radiological examination showed swelling in the cervical cord. This case report strongly focuses on the radiological findings of NMO.

## Introduction

Neuromyelitis optica (NMO), also known as Devic’s disease, is a rare and severe autoimmune disorder that primarily affects the optic nerves and spinal cord [1]. It was commonly misdiagnosed as multiple sclerosis (MS), but studies have shown that it has distinct pathological clinical characteristics. This disorder was first discovered by Devic and Gault in 1894, but it was not until the twenty-first century that its underlying pathogenesis and diagnostic criteria were revealed [2].

In the past, NMO was thought to be a more advanced form of MS; however, unlike MS, most forms of NMO have autoantibodies against either aquaporin-4 (AQP4) channels or myelin oligodendrocyte glycoprotein [1,3]. One of the hallmarks of NMO, but not specifically, is the presence of specific autoantibodies targeting the AQP4 water channel protein, which is in the astrocyte end-feet in the central nervous system and is adjacent to the cerebrospinal fluid [4]. These autoantibodies cause damage and inflammation to the optic nerves and spinal cord, resulting in blindness, paralysis, and chronic disability. In addition, they cause symptoms such as myelitis, extreme optic neuritis, and area postrema syndrome, which consists of uncontrollable vomiting and hiccups [3].

The diagnosis of NMO requires the fulfillment of clinical and radiological criteria, as well as with or without the detection of AQP4 antibodies (AQP4-Abs) in the blood or cerebrospinal fluid. The treatment of NMO involves immunosuppressive therapies, plasma exchange, and supportive care [3]. The prognosis of NMO is variable, with some patients experiencing frequent relapses and disabilities, while others have a more benign course.

Despite the advances in understanding the pathogenesis and treatment, much remains unknown about this rare condition. The following case report supports radiological evidence findings of this condition.

## Case presentation

A 25-year-old African American male initially presented with a complaint of blurry vision in his right eye, progressively getting worse. He described his vision as a black spot in the right eye and pain elicited by an upward gaze. He also complained of intermittent tingling in both hands and feet. Symptoms started six months ago and have been relapsing and remitting. He denies any flashing lights or floaters and had no weakness, numbness, or tingling in his extremities at the time of presentation.

**Figure 1:**
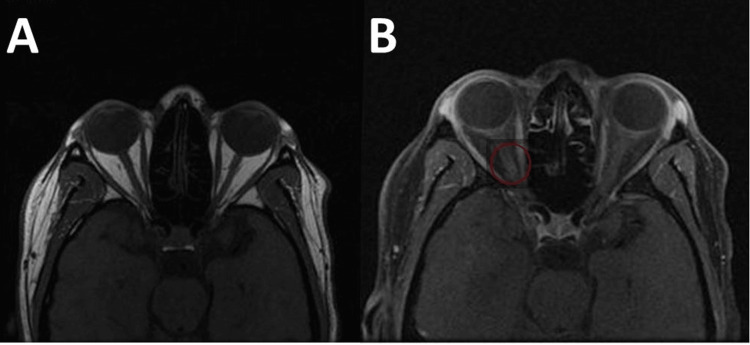
Axial T1 MRI of the orbits at the level of the optic nerve: (A) precontrast, unremarkable; (B) postcontrast, asymmetric hyperenhancement of the right optic nerve compared with the left side (circled). MRI, magnetic resonance imaging

On physical exam, the patient showed loss of central vision in his right eye, with peripheral vision spared. On initial presentation, vision in his right eye was 20/25 and continued to go down to count fingers at 2 feet the next day. His left eye was normal and 20/20 vision. Pupils were round and reactive. On visual field examination, the right eye showed decreased vision on the nasal side superiorly and centrally. The left eye has a full and normal visual field. A slit lamp examination showed normal findings. He was unable to see any color plates; red appeared dimmer on the right eye than on the left. On optical coherence tomography, the right eye showed a swelling superiorly in the retina nerve fiber layer. Additionally, a computed tomography (CT) of the head showed a small degree of superior swelling, suggesting increased pressure behind the nerve head. This was clearly seen when axial T1 MRI was performed pre- and postcontrast of the optic nerve (Figure 1).

**Figure 2:**
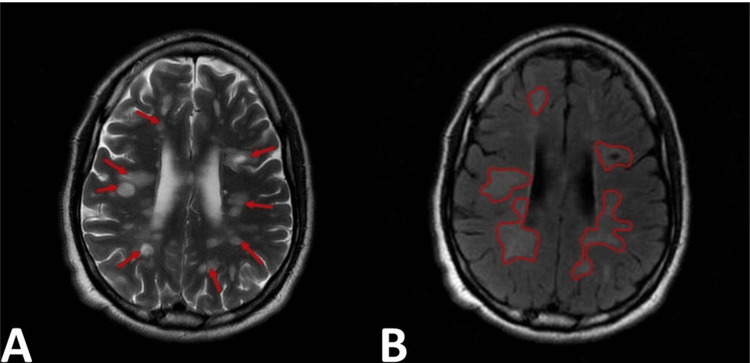
Axial T2 MRI of the brain at the level of the ventricles: (A) innumerable patchy foci of bright signal intensity throughout the white matter (red arrows); (B) patchy foci of edema associated with the lesions (outlined). MRI, magnetic resonance imaging

**Figure 3:**
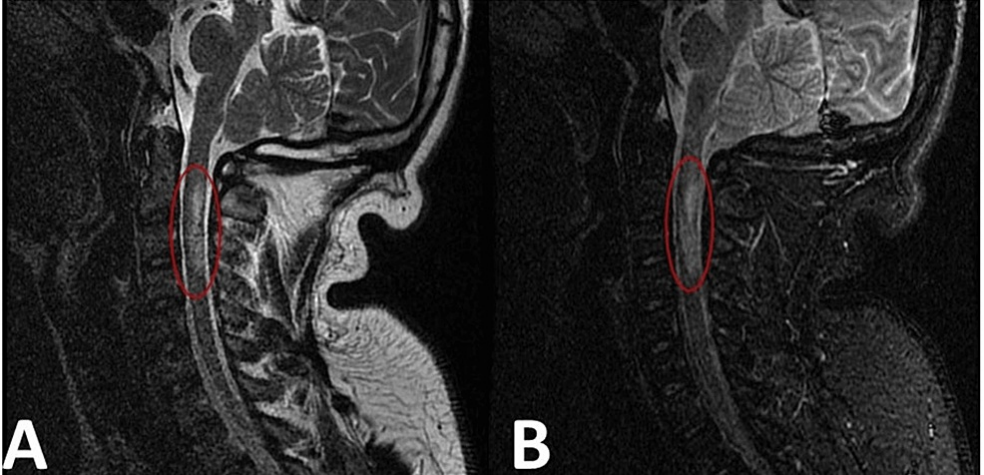
(A) Sagittal T2 MRI of the cervical spine demonstrating a large patchy area of increased T2 signal within the spinal cord at the level of C2-C4 (circled); (B) sagittal T2 STIR MRI of the cervical spine demonstrating a large area of edema within the upper cervical spinal cord from the levels of C2-C4 corresponding to the area of increased T2 signal in (A) (circled). STIR, short tau inversion recovery; MRI, magnetic resonance imaging

Initially, the diagnosis was thought to be retinal detachment and later determined to be posterior optic neuritis. He was started on intravenous methylprednisolone. MRI of the head and C-spine showed multiple white matter lesions varying in appearance, diffusely throughout the brain parenchyma (Figure 2). A large focus of these lesions is pericallosal in nature. The majority were bright on T2 and fluid-attenuated inversion recovery (FLAIR) sequences, some of which were dark on the T1 images. A few lesions, most notably in the right parietal lobe, demonstrated an internal high T2 signal, which suppressed FLAIR sequences and was dark on T1-weighted images. Another subset of lesions was ring-enhancing on the postcontrast T1 sequences. For example, in the left occipital lobe, there was a lesion measuring 1.3 cm × 1.1 cm and another lesion measuring 0.6 cm in the left frontal lobe. There was no evidence of blooming on the gradient echo sequence to suggest hemorrhage. No significant mass effect or edema was identified. Additionally, there were bright signal intensity and swelling in the upper cervical cord representing a possible diagnosis of NMO (Figure 3). The patient was tested seronegative for AQP4 IgG Abs. He was discharged on oral prednisone 60 mg per day. 

## Discussion

NMO, also known as Devic's disease, is a rare condition associated with systemic autoimmune disorders, including Sjogren's syndrome, systemic lupus erythematosus, thyroid diseases, and myasthenia gravis [5]. NMO is a spectrum disorder that is subdivided into two categories based on the presence of AQP4-Abs (seronegative and seropositive). Previously, it was thought to be a monophasic disorder such as inflammatory episodes of the optic nerve and spinal cord that occur for a period and does not recur after the treatment. But the current guidelines indicate the risk of multiple recurrences after remission of the disease for weeks, months, or years. 

In the initial stage of the disease, it can be misdiagnosed as MS [6]. As it was previously considered a variant of MS, the discovery of AQP4-Ab grouped it as a separate diagnosis with a different entity [6,7]. NMO usually presents with myelitis or optic neuritis as the initial manifestation of the disease. Optic neuritis is the inflammation of the optic nerve that leads to pain in the affected eye which is followed by loss of visual acuity in the affected eye. It is usually unilateral, however, sometimes both eyes can be affected at the same time [6]. NMO can also cause transverse myelitis, which is inflammation of the spinal cord. Transverse myelitis is usually unilateral and presents on one side of the body with loss of autonomic, sensory, and motor functions, including bowel and bladder functions. These symptoms cannot be distinguished from classic idiopathic transverse myelitis [8]. 

Based on clinical symptoms, NMO is difficult to distinguish from MS because both disorders present with transverse myelitis and optic neuritis. MRI is the modality of choice. According to the literature, imaging studies reveal longitudinally extensive transverse myelitis with T1 hypointensity, periependymal brainstem changes, and perivenous white matter lesions [9]. All the affected parts such as the brain, orbit, and spinal cord should be investigated under MRI. In NMO, orbits may exhibit typical features of optic neuritis, i.e., hyperintense optic nerve, atrophy of optic nerve in chronic stages of the disorder, and bilateral involvement of optic nerve with the abnormal extension of the signal [7]. The intracranial appearance of the brain is usually normal; however, some abnormalities have been noted in seropositive NMO that include, a marbled pattern of the corpus callosum, deep punctate white matter lesions, extensive longitudinal lesions in the corticospinal tract, and more than 3 cm diameter white matter lesions in the hemisphere that are radially oriented that gives a spilled-ink appearance of the lesion. It was also estimated that there is a positive correlation between the segmental length of spinal cord lesions and the expected disability [10]. In the spinal cord, more than three consecutive vertebral segments are affected, creating a longitudinally extensive spinal cord lesion. In the acute phase, the spinal cord is swollen. The lesions are bright and spotted, which are characteristics of the NMO [7]. In our case, there is the patient classic presentation of NMO with white matter lesions and swelling in the C-spine extending to brain parenchyma on radiological findings.

Our patient was diagnosed with seronegative NMO, and recent studies have shown that rituximab is an effective therapy for resistant or seronegative NMO patients, and some consider it the first-line treatment [11,12]. Traditional treatment for acute attacks in seronegative NMO consists of intravenous corticosteroids with or without plasma exchanges, with a recovery rate of nearly 60%. NMO is strongly associated with relapse (high AQP4-Ab titers), necessitating the use of immunosuppressive maintenance therapy. With or without low-dose oral corticosteroids, azathioprine is regarded as moderately effective in reducing the frequency of attacks. 

## Conclusions

Our study focused on the radiological significance of NMO. It is based on a young African American male, with the possibility of NMO as it was revealed in the radiological findings, i.e., white matter lesions and swelling in the C-spine extending to the brain parenchyma. Although serological findings were negative, the treatment was based on intravenous corticosteroids with or without plasmapheresis. This research focused only on the radiological aspect of the disease, but further research is needed to explore the clinical aspect of the disease.
